# Supplementary material for: ETV2 Mediated Differentiation of Human Pluripotent Stem Cells Results in Functional Endothelial Cells for Engineering Advanced Vascularized Microphysiological Models
Source: Adv Healthc Mater. 2026 Mar 12;15(19):e04849. doi: 10.1002/adhm.202504849 (PMC13206403; doi:10.1002/adhm.202504849)
Supplement: Supplementary file 1 — Supporting File: adhm71025‐sup‐0001‐SuppMat.docx. [file ADHM-15-0-s001.docx]

((Supporting Information can be included here using this template))

Supporting Information

ETV2 mediated differentiation of human pluripotent stem cells results in functional endothelial cells for engineering advanced vascularized microphysiological models

Shun Zhang*^#^, Zhengpeng Wan^#^, Lei Wang, Caihong Wu, Junkai Zhang, Sarah Spitz, Xun Wang, Marie A. Floryan, Mark F Coughlin, Francesca M. Pramotton, Liling Xu, Ron Weiss, Roger D. Kamm*

S. Zhang, C. Wu, J. Zhang

State Key Laboratory of Organ Regeneration and Reconstruction, Institute of Zoology, Chinese Academy of Sciences, Beijing 100101, China

Beijing Institute for Stem Cell and Regenerative Medicine, Beijing 100101, China

Z. Wan, L. Wang, X. Wang, M. F. Coughlin, F. Pramotton, R. Weiss, R. D. Kamm

Department of Biological Engineering, Massachusetts Institute of Technology, Cambridge, MA, 02139, USA

Z. Wan

Department of Biomedical Engineering, Michigan Technological University, Houghton, MI, USA

Health Research Institute, Michigan Technological University, Houghton, MI, USA

M. A. Floryan, R. D. Kamm

Department of Mechanical Engineering Massachusetts Institute of Technology Cambridge, MA 02139, USA

L. Wang

Bioengineering Department, Northeastern University, Boston, MA, 02115, USA

L. Xu

Ragon Institute of Mass General Brigham, MIT and Harvard, Cambridge, MA, 02139, USA

L. Wang, R. Weiss

Synthetic Biology Center, Massachusetts Institute of Technology, Cambridge, MA, 02139, USA

*corresponding authors, ^#^contributed equally to this work

E-mail: shunzhang@ioz.ac.cn, rdkamm@mit.edu


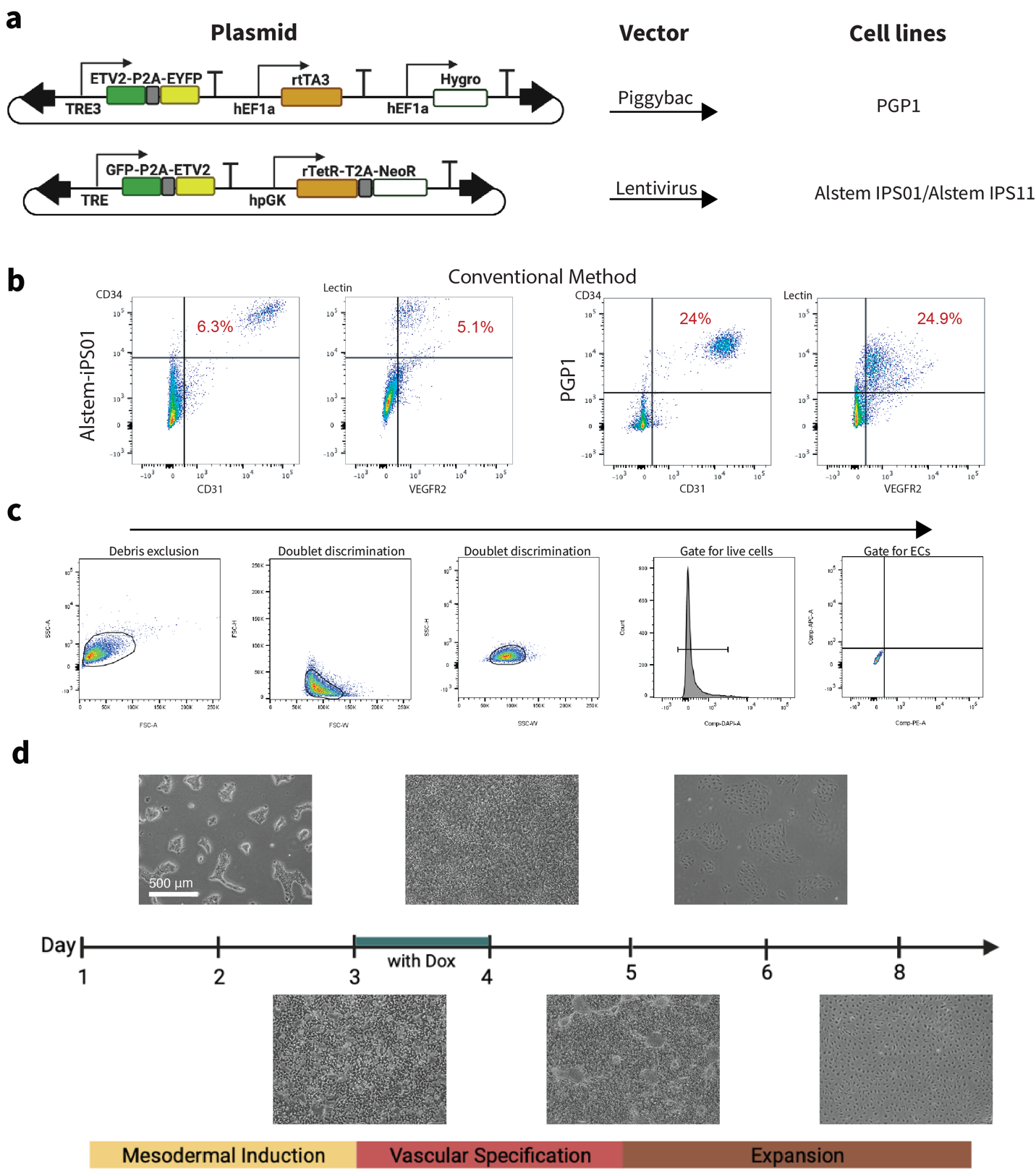


Figure S1. Differentiation of h-iECs from h-iPSCs with engineered inducible ETV2 with optimized protocol. (a) Generation of various h-iPSC lines with inducible ETV2 using Piggybac or lentivirus. (b) Differentiation efficiency of h-iPSCs into CD31^+^/CD34^+^/VEGFR2^+^/ UEA-I ^+^ h-iECs with conventional two step method by flow cytometry. (c) Gating strategy for ECs. (d) Bright field images showing differentiation process of h-iECs from h-iPSCs with engineered inducible ETV2. Scale bar is 500 μm.


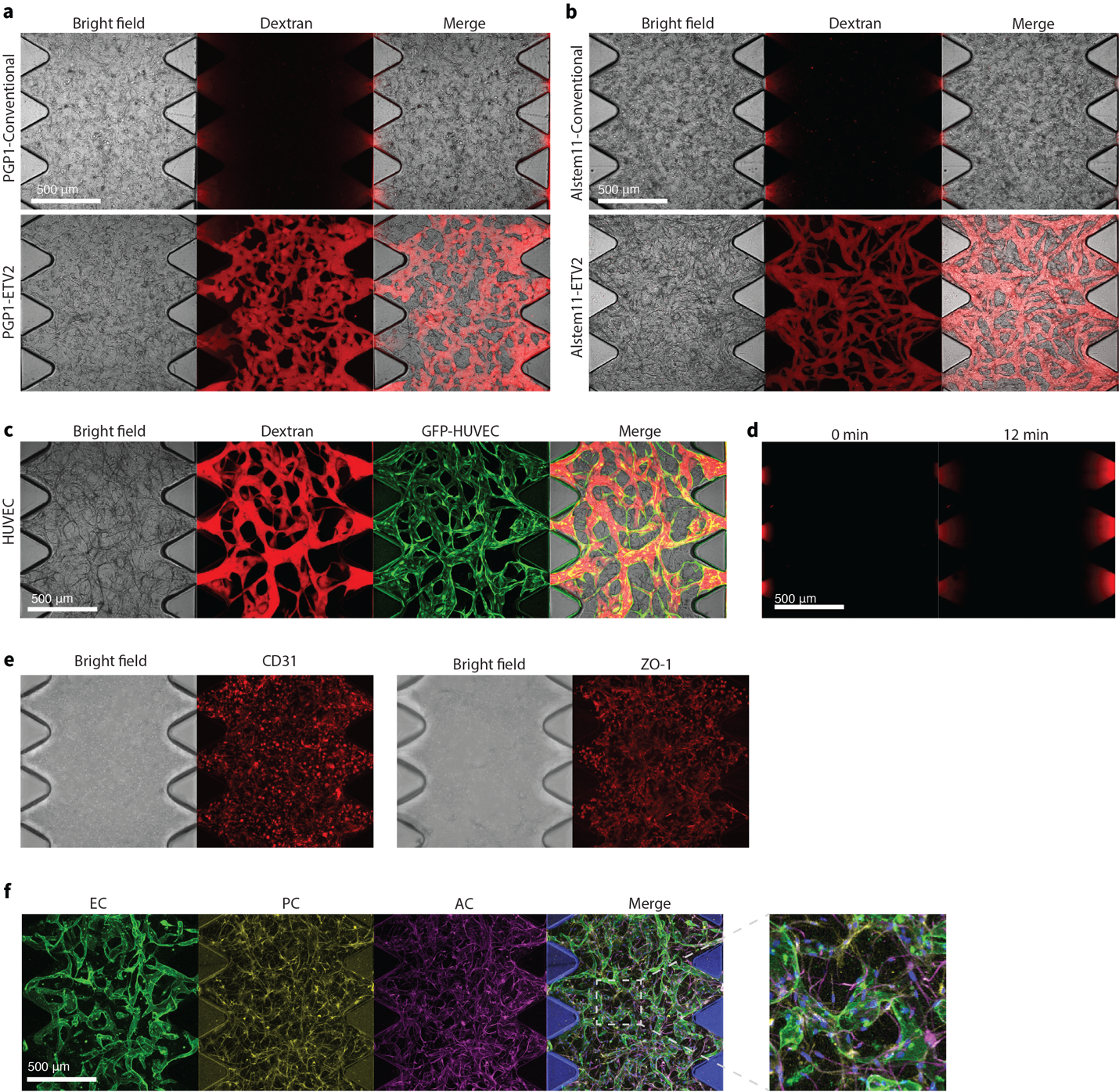


Figure S2. Formation of functional MVNs with h-iECs differentiated through transient activation of ETV2. (a) Representative images of MVNs made of h-iECs on day 7. h-iECs were differentiated from PGP1 h-iPSCs using either conventional method or optimized protocol with ETV2 activation. Perfusion test was performed with 40 kDa Texas Red dextran (red). (b) Representative images of MVNs made of h-iECs on day 7. h-iECs were differentiated from Alstem iPS11 h-iPSCs using either conventional method or optimized protocol with ETV2 activation. Perfusion test was performed with 40 kDa Texas Red dextran (red). (c) Representative images of MVNs formed using HUVECs and HLFs at day 7. Red: 40 kDa Texas Red dextran, Green: GFP HUVECs. (d) Representative images of perfusing empty fibrin gel with 40 kDa Texas Red dextran. Images were acquired at 0 and 12 mins. (e) Representative images of day 7 MVNs made of h-iECs that were differentiated from PGP1 h-iPSCs using conventional method. ECs were stained with CD31 and ZO-1. (f) Immunoﬂuorescence staining for brain specific MVNs made of h-iECs, human brain pericytes (PCs), and astrocytes (ACs), with zoomed-in view. Green: CD31 staining for h-iECs, magenta: S-100b staining for ACs, yellow: PDGFR staining for PCs. All scale bars are 500 μm.


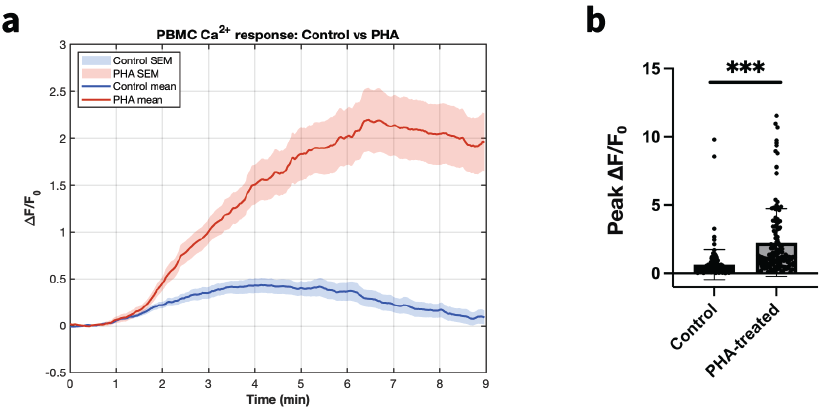


Figure S3. Live-cell Ca²⁺ imaging of PBMCs loaded with fluorescent Ca²⁺ indicator under control conditions or following phytohemagglutinin (PHA) stimulation. (a) Time courses of single-cell Ca²⁺ responses expressed as ΔF/F₀, shown as mean ± SEM across cells, with time displayed in minutes. Shaded areas indicate SEM. (b) Box plots of the maximal Ca²⁺ response per cell (peak ΔF/F₀) for control and PHA-treated groups, illustrating the distribution and shift toward higher Ca²⁺ signaling upon PHA stimulation. Significance was calculated with t-tests. ***P < 0.01.


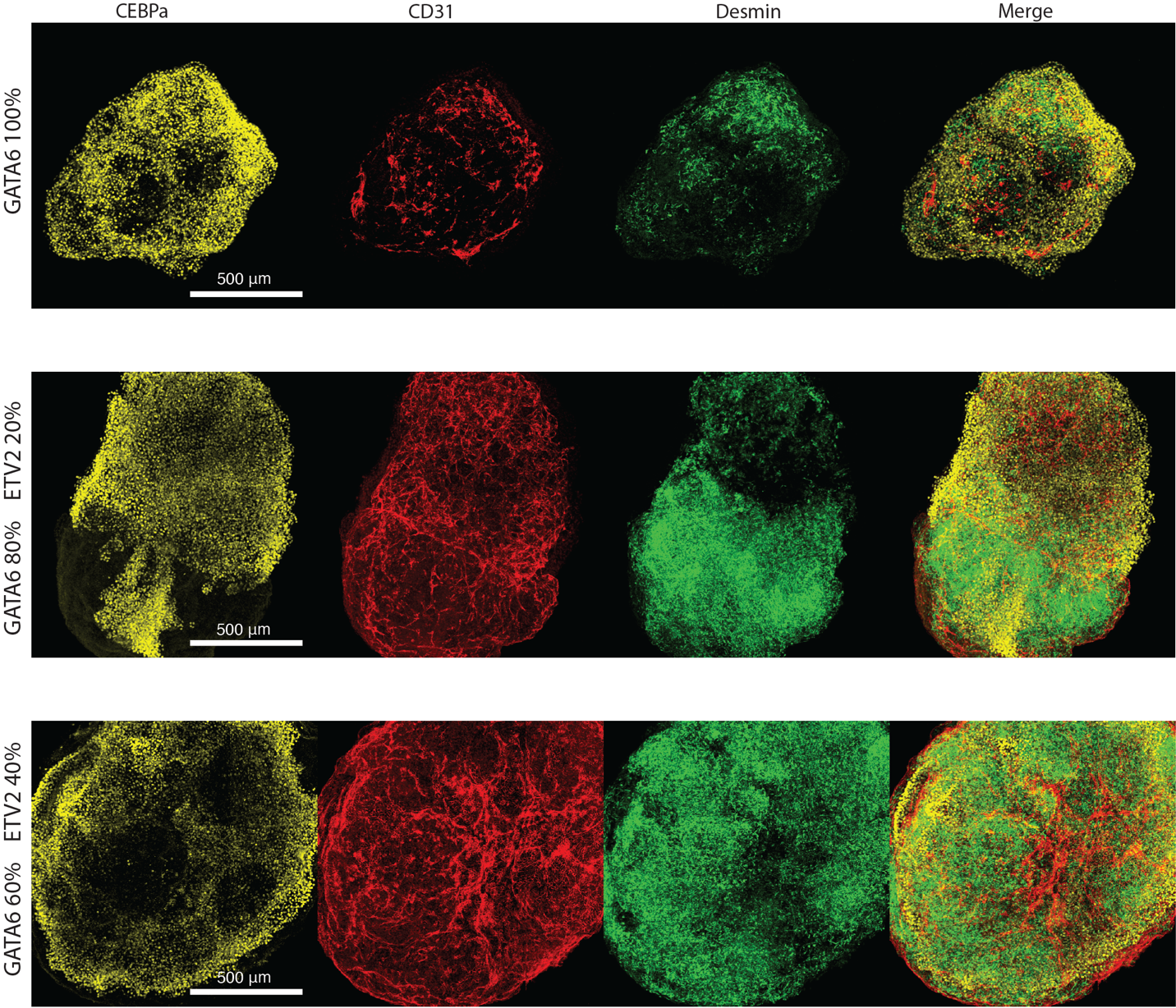


Figure S4. Immunoﬂuorescence staining for liver organoids formed by pooling different ratios of PGP1-GATA6 and PGP1-ETV2. Yellow: CEBPa staining for hepatocytes, Red: CD31 staining for ECs, Green: Desmin staining for stellate cells. Scale bar is 500 μm.


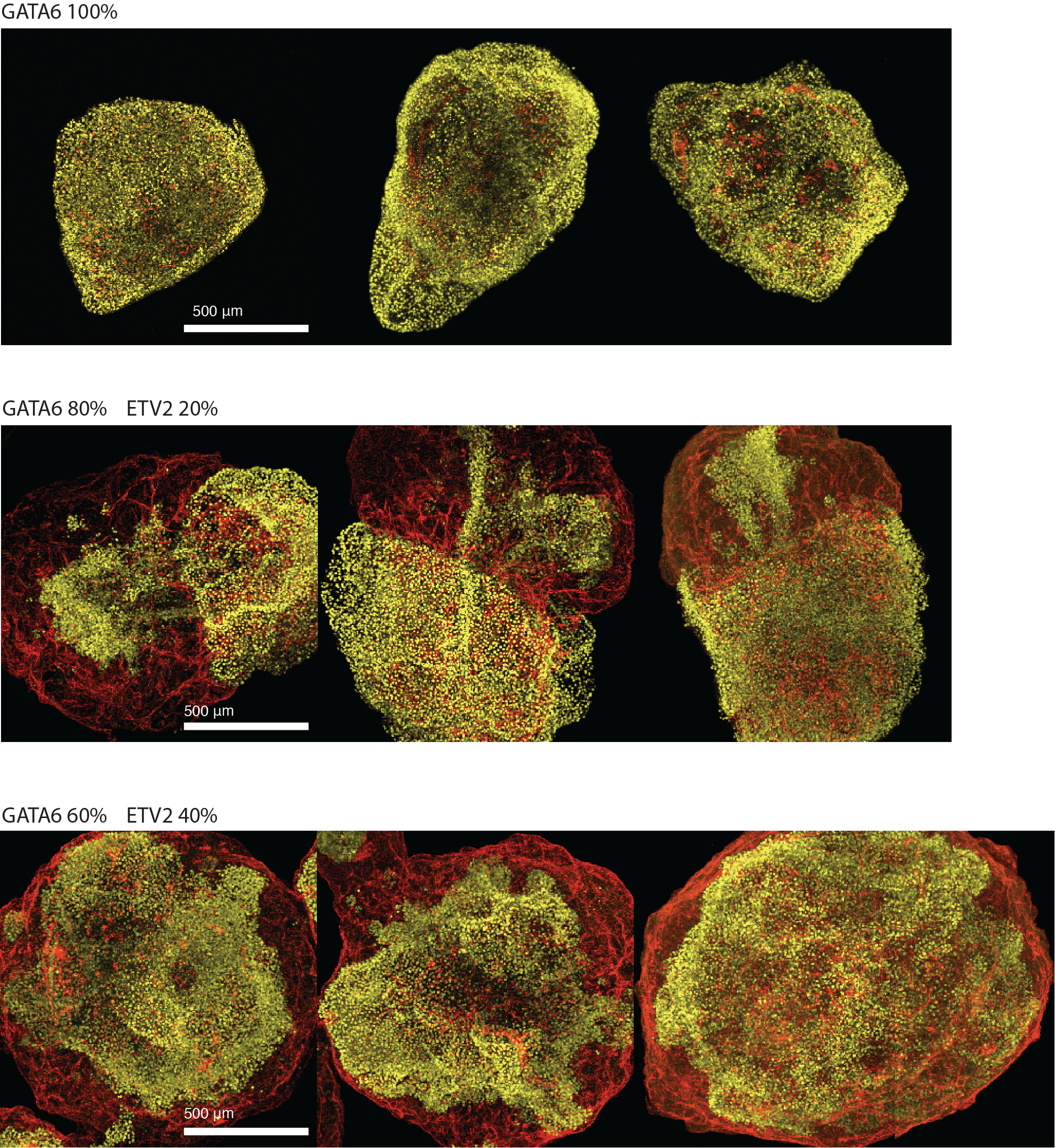


Figure S5. Spatial arrangements of ECs and hepatocytes within liver organoids formed by pooling different ratios of PGP1-GATA6 and PGP1-ETV2. Yellow: CEBPa staining for hepatocytes, Red: CD31 staining for ECs. Scale bars are 500 μm.


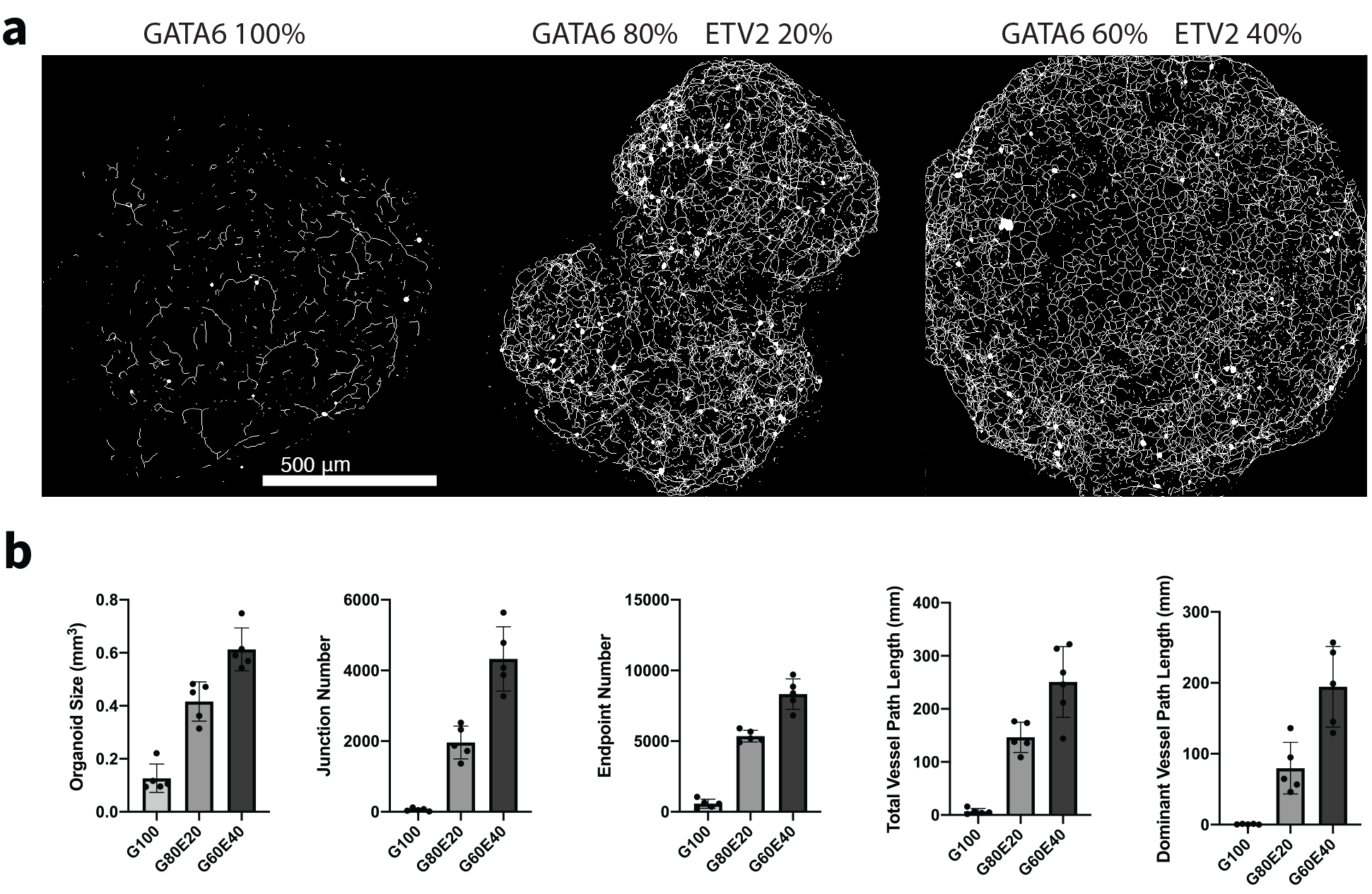


Figure S6. Characterization of vascular networks within liver organoids formed by pooling different ratios of PGP1-GATA6 and PGP1-ETV2. (a) 3D skeletons of vascular networks within liver organoids. Scale bar is 500 μm. (b) Detailed characterization of morphological parameters of vascular networks within liver organoids formed by pooling different ratios of PGP1-GATA6 and PGP1-ETV2.
